# Idiopathic Atrial Flutter With Tachycardia‐Induced Cardiomyopathy Detected at a Routine Infant Health Checkup: A Case Report

**DOI:** 10.1155/crpe/4811651

**Published:** 2026-08-21

**Authors:** Toshinobu Ifuku, Fumito Wakamatsu, Keigo Nakatani

**Affiliations:** ^1^ Department of Pediatrics, Miyazaki Prefectural Miyazaki Hospital, Miyazaki, Japan, kenritsu-miyazakibyouin.jp; ^2^ Department of Pediatrics, Miyazaki Prefectural Nichinan Hospital, Miyazaki, Japan

**Keywords:** arrhythmia-induced cardiomyopathy, atrial flutter, auscultation, infant

## Abstract

Idiopathic atrial flutter (AFL) is an uncommon supraventricular tachyarrhythmia in early infancy. When sustained, AFL may lead to tachycardia‐induced cardiomyopathy (TIC), a potentially reversible cause of ventricular dysfunction after rhythm control. We report a 3‐month‐old boy in whom severe TIC was detected incidentally at a routine infant health checkup despite minimal symptoms. The examining pediatrician noted persistent age‐inappropriate tachycardia and referred the infant urgently. On admission, the ventricular rate fluctuated between 160 and 240 beats/min, likely reflecting variable atrioventricular conduction. Chest radiography showed marked cardiomegaly. Echocardiography revealed a severely dilated left ventricle (left ventricular end‐diastolic dimension 41 mm; Z‐score +7.3) with diffuse hypokinesis and markedly reduced systolic function (left ventricular ejection fraction approximately 20%), consistent with advanced TIC. Adenosine triphosphate transiently induced atrioventricular block and unmasked typical flutter waves, confirming AFL. Synchronized direct‐current cardioversion (1 J/kg) immediately restored sinus rhythm, followed by standard heart failure therapy. Rapid reverse remodeling was documented quantitatively, with regression of cardiomegaly and normalization of ventricular size and function. The infant was discharged on Day 25 and remained well without recurrence at follow‐up. This case highlights that AFL in infancy can be clinically subtle while causing profound TIC and underscores the importance of careful heart rate assessment during infant health supervision.

## 1. Introduction

Idiopathic atrial flutter (AFL) is an uncommon supraventricular tachyarrhythmia in infancy. Most cases are diagnosed in the perinatal period, and the long‐term prognosis is generally favorable once sinus rhythm is restored [1].

Tachyarrhythmias can also trigger a reversible dilated cardiomyopathy referred to as tachycardia‐induced cardiomyopathy (TIC) [2, 3]. However, infants with sustained tachycardia cannot describe symptoms, and clinical manifestations are often nonspecific; consequently, diagnosis may be delayed until overt heart failure or cardiogenic shock develops [4, 5].

Detection during routine health supervision before decompensation is uncommon and provides a practical lesson for primary care. We report an infant in whom idiopathic AFL with TIC was detected incidentally at a routine health checkup.

## 2. Case Presentation

A male infant was born at term after an uneventful pregnancy and delivery. He was the first child of healthy, nonconsanguineous parents. Prenatal ultrasonography showed no fetal arrhythmia or structural heart disease. He was delivered by caesarean section at 40 + 2 weeks’ gestation because of cephalopelvic disproportion, with a birth weight of 4020 g and Apgar scores of 8 and 10 at 1 and 5 min, respectively. Newborn metabolic screening was normal. During his discharge from the maternity ward and at his 1‐month and 2‐month checkup, no problems with his overall condition, including abnormal tachycardia, were noted.

At 111 days of age, he attended a scheduled municipal health checkup. The examining pediatrician detected marked tachycardia on auscultation and pulse palpation, estimating a heart rate greater than 200 beats/min, and referred the infant to our hospital the same day. The parents reported that he was active, feeding well, and gaining weight appropriately (8700 g), although they had retrospectively noticed occasional irregular breathing after feeding over the past few days. There was no history of intercurrent infection. He had not taken any medications or supplements. Family history was negative for congenital heart disease, arrhythmias, sudden cardiac death, or cardiomyopathy.

On admission, the infant appeared well and showed no obvious distress. He was afebrile, with a blood pressure of 89/45 mmHg, a respiratory rate of 32/min, and an SpO_2_ of 100% on room air. The ventricular rate fluctuated between 160 and 240 beats/min at rest. The heart rate did not vary with rest, activity, or crying. There were no heart murmurs or gallop rhythms. Hepatomegaly was indistinct, and capillary refill time was mildly prolonged at 3 s.

**FIGURE 1:**
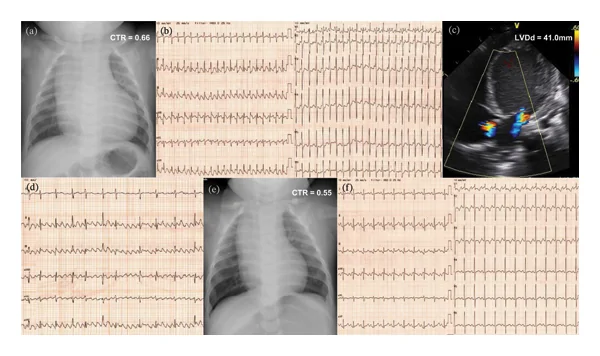
Clinical images showing the course of diagnosis and treatment. (a) Chest radiograph on admission showing massive cardiomegaly (cardiothoracic ratio [CTR] 0.66) without pulmonary congestion. (b) Twelve‐lead electrocardiogram (ECG) on admission showing regular narrow QRS tachycardia (heart rate approximately 240 bpm) with obscure P waves. (c) Transthoracic echocardiography (apical four‐chamber view) on admission revealing a severely dilated left ventricle with mild mitral regurgitation. (d) ECG during rapid intravenous administration of adenosine triphosphate (0.2 mg/kg). Transient atrioventricular block unmasks typical “sawtooth” flutter waves, confirming atrial flutter. (e) Chest radiograph on Day 12 showing significant improvement in cardiomegaly (CTR 0.55). (f) ECG after synchronized electrical cardioversion showing restoration of sinus rhythm without evidence of ventricular pre‐excitation, including no delta waves, short PR interval, or QRS widening. LVDd, left ventricular end‐diastolic dimension.

Chest radiography showed massive cardiomegaly with a cardiothoracic ratio of 0.66, without pulmonary congestion (Figure 1a). A 12‐lead electrocardiogram (ECG) showed narrow QRS tachycardia with indistinct atrial activity (Figure 1b).

Echocardiography demonstrated a severely dilated left ventricle (LV) with a left ventricular end‐diastolic dimension (LVDd) of 41 mm (Z‐score +7.3) and diffuse hypokinesis, with a left ventricular ejection fraction (LVEF) of approximately 20% and mild mitral regurgitation (Figure 1c). No obvious cardiovascular malformations were identified.

Laboratory testing showed markedly elevated N‐terminal probrain natriuretic peptide (NT‐proBNP, 19,927 pg/mL). Cardiac troponin T was mildly elevated (0.27 ng/mL; reference < 0.1), whereas creatine kinase and inflammatory markers were within normal ranges (Table 1). Microbiological tests, including polymerase chain reaction analyses of serum, pharyngeal mucus, feces, and urine, did not identify a clear pathogen. Metabolic screening for mitochondrial or lysosomal storage diseases was negative.

**TABLE 1:** Initial laboratory data.

**Peripheral blood tests**		**(Normal range)**

WBC	11.80	3.30–8.60 × 103/μL
Neu	22.0	40.0%–70.0%
Ly	59.5	18.0%–53.0%
RBC	4.80	4.35–5.55 × 106/L
Hb	11.9	13.7–16.8 g/dL
Ht	37.4	40.7%–50.1%
MCV	78.0	83.6–98.2 fL
Platelet	413	158–348 × 103/μL

**Coagulation study**		**(Normal range)**

PT	13.3	10.0–13.5 s
APTT	18.5	24.0–40.0 s
PT‐INR	1.07	0.90–1.10
D‐Dimer	0.37	0.0–1.0 μg/mL

**Biochemistry tests**		**(Normal range)**

TP	5.9	6.6–8.1 g/dL
Alb	4.2	4.1–5.1 g/dL
BUN	10.1	8.0–20.0 mg/dL
Cr	0.28	0.65–1.07 mg/dL
CRP	0.04	0–0.14 mg/dL
Na	141	138‐145 mEq/L
K	5.1	3.6–4.8 mEq/L
Cl	107	101‐108 mEq/L
Ca	10.6	8.8–10.1 mg/dL
T‐Bil	0.59	0.4–1.5 mg/dL
AST	32	13–30 IU/L
ALT	20	10–42 IU/L
CPK	97	59–248 IU/L
LDH	300	124–222 IU/L
γ‐GTP	21	13–64 IU/L
Glucose	77	73–109 mg/dL
NT‐proBNP	19,927	0–55 pg/mL
cTnT	0.27	0–0.1 ng/mL
TSH	2.57	0.35–4.94 μU/mL
FT4	1.13	0.70–1.48 ng/dL

**Vein blood gas analysis**		**(Normal range)**

pH	7.340	7.350–7.450
PCO_2_	43.2	35.0–45.0 mmHg
PO_2_	38.0	75.0–100.0 mmHg
HCO_3_	22.7	20.0–26.0 mmol/L
BE	−2.5	−3.0‐3.0 mmol/L
Lactate	2.40	0.50–2.00 mmol/L

*Note:* Neu, neutrophils; Ly, lymphocytes; Hb, hemoglobin; Ht, hematocrit; Alb, albumin; Cr, creatinine; Na, sodium; K, potassium; Cl, chlorine; Ca, calcium; T‐Bil, total‐bilirubin; AST, aspartate aminotransferase; ALT, alanine aminotransferase. CPK, creatinine phosphokinase; LDH, lactase dehydrogenase; γ‐GTP, γ‐glutamyl transpeptidase; NT‐proBNP, N‐terminal probrain natriuretic peptide; cTnT, Cardiac troponin‐T. FT4, free thyroxine.

Abbreviations: APTT, activated partial thromboplastin time; BE, base excess; BUN, blood urea nitrogen; CRP, C‐reactive protein; MCV, mean corpuscular volume; PT, prothrombin time; PT‐INR, prothrombin time‐international normalized ratio; RBC, red blood cell; TP, total protein; TSH, thyroid‐stimulating hormone; WBC, white blood cell.

To clarify the diagnosis, adenosine triphosphate (ATP) was administered rapidly intravenously (0.2 mg/kg). Transient atrioventricular block unmasked typical “sawtooth” flutter waves (Figure 1d), confirming AFL. Given the severe LV dysfunction despite minimal symptoms and the risk of clinical deterioration, we prioritized prompt rhythm control. Following intravenous administration of 8 μg of fentanyl and 0.8 mg of midazolam, synchronized electrical cardioversion (1 J/kg) was performed and immediately restored sinus rhythm. A 12‐lead ECG obtained after cardioversion documented sinus rhythm without findings suggestive of ventricular pre‐excitation, including a short PR interval, delta waves, or QRS widening (Figure 1f).

After cardioversion, heart failure therapy was initiated with intravenous milrinone (0.25 μg/kg/min, later tapered to 0.125 μg/kg/min) and furosemide, along with fluid restriction. Oral heart failure medications were introduced and uptitrated during the hospital stay, including furosemide and spironolactone, low‐dose aspirin (3–5 mg/kg/day), and carvedilol, which was started at 0.05 mg/kg/day and increased to 0.3 mg/kg/day as tolerated. Aspirin was temporarily administered as antiplatelet therapy because the patient presented with severe left ventricular enlargement and marked impairment of systolic function, raising concerns about the formation of intraventricular thrombi until ventricular function recovered. No intracardiac thrombus was detected on echocardiography, and blood tests revealed no signs of coagulation disorders; therefore, systemic anticoagulation therapy was not administered. After left ventricular systolic function normalized, aspirin administration was discontinued during a follow‐up outpatient visit following discharge. No antiarrhythmic drug was given for recurrence prophylaxis because sinus rhythm remained stable.

**FIGURE 2:**
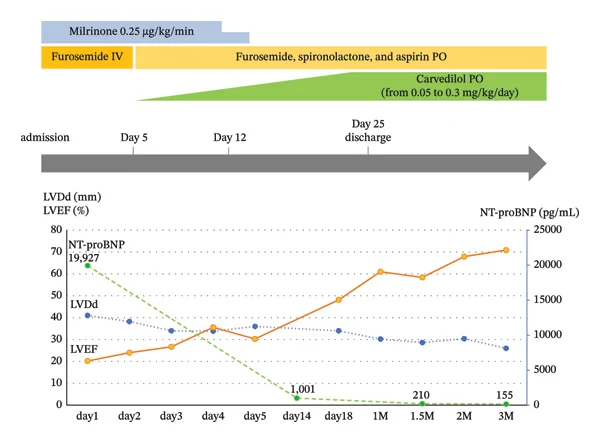
Timeline of clinical course and serial changes in cardiac function after rhythm control. The upper panel summarizes the treatment course after admission. Key time points (admission, Days 5 and 14, and discharge) are indicated. The lower panel shows serial echocardiographic measurements of left ventricular end‐diastolic dimension and left ventricular ejection fraction plotted on the left *y*‐axis, and NT‐proBNP plotted on the right *y*‐axis. LVDd, left ventricular end‐diastolic dimension; LVEF, left ventricular ejection fraction; NT‐proBNP, N‐terminal pro‐B‐type natriuretic peptide; IV, intravenous; PO, per os.

Cardiac function improved steadily after restoration of sinus rhythm. Serial chest radiographs showed regression of cardiomegaly, with the cardiothoracic ratio decreasing from 0.66 on admission to 0.61 by Day 8 and 0.55 by Day 12 (Figure 1e). Echocardiography on Day 18 showed improvement in LVDd to 34 mm (Z‐score +3.4), with normalization of LVEF (> 55%). Mitral regurgitation was also confirmed to have resolved. He was discharged home on Day 25 with carvedilol, diuretics, and low‐dose aspirin. At 10‐month follow‐up, he remained asymptomatic with age‐appropriate growth and development, and both echocardiographic findings and NT‐proBNP levels had normalized. Repeated Holter monitoring detected no clinically significant arrhythmias, including AFL. The clinical course, treatment details, and test data from hospitalization through the early postdischarge phase are presented in Figure 2.

## 3. Discussion

This case illustrates an uncommon presentation of idiopathic AFL in early infancy, detected incidentally at a well‐baby examination yet already complicated by severe TIC. It highlights several points regarding the natural history of infant AFL, the pathophysiology of TIC, and the role of primary care in detecting arrhythmias.

AFL in infants is often considered idiopathic when no structural heart disease or postoperative substrate is present. Large series have shown that infant AFL frequently presents in the first days of life, and many patients are asymptomatic at diagnosis [1, 2]. Texter et al. [1] reported 50 infants with AFL without prior cardiac surgery; approximately 80% were asymptomatic at diagnosis, and most presented within 48 h of birth. In contrast, AFL onset beyond the neonatal period is less common and may be associated with a higher risk of congestive heart failure.

Although our patient had subtle parental observations of irregular breathing, he had no overt signs of heart failure at the time of the health check. This underscores that sustained tachyarrhythmias in infants can remain clinically silent until myocardial dysfunction becomes advanced. Notably, the ventricular rate fluctuated between 160 and 240 beats/min, which is compatible with variable atrioventricular conduction during AFL, including intermittent changes in conduction ratio. This variability may contribute to delayed recognition if tachycardia is intermittently less striking. The discrepancy between clinical appearance and disease severity is therefore a key pitfall in infant tachyarrhythmias.

Previous series suggest that isolated AFL in neonates and young infants has a low recurrence rate after restoration of sinus rhythm. Casey et al. [6] reported no recurrence of AFL during a median follow‐up of 23 months in 25 neonates, and Peng et al. [7] similarly reported no recurrence during 0.5–7 years of follow‐up in seven patients presenting within the first 3 months of life. In the larger infant series, AFL without other arrhythmias was associated with a low risk of recurrence; recurrent cases were reported mainly among patients with additional arrhythmias, congestive heart failure, valvular disease, or electrolyte abnormalities, and often required antiarrhythmic therapy [1, 8, 9]. Therefore, long‐term antiarrhythmic prophylaxis may not be routinely required after successful cardioversion in isolated infantile AFL. However, ongoing rhythm surveillance remains important, particularly when AFL recurs or when pre‐excitation, frequent atrial ectopy, structural heart disease, or another supraventricular tachycardia substrate is suspected. Because AFL in infants may coexist with accessory pathway‐mediated tachycardia, careful review of the postcardioversion 12‐lead ECG is useful to assess for WPW syndrome. In our patient, the absence of pre‐excitation, recurrent AFL, or clinically significant ectopy on follow‐up Holter monitoring.

TIC is a well‐recognized yet underdiagnosed cause of dilated cardiomyopathy, resulting from persistent tachyarrhythmias such as atrial fibrillation, AFL, or incessant supraventricular tachycardia [5, 10]. Experimental and clinical data show that sustained high heart rates can lead to ventricular dilation, reduced systolic function, and neurohormonal activation; importantly, these changes are at least partly reversible once the arrhythmia is controlled [5]. Pediatric data on AFL‐induced TIC remain limited. Recent case reports have described infants presenting with overt heart failure, marked LV dysfunction, and AFL, followed by normalization of function after cardioversion and rate control [4, 5, 10]. Our patient had minimal symptoms and appeared clinically stable, yet showed profound LV dilation and dysfunction at presentation.

The differential diagnosis for this presentation needs to be considered at two levels: persistent narrow QRS tachycardia and secondary ventricular dysfunction. Persistent tachycardia may reflect sinus tachycardia caused by fever, dehydration, anemia, sepsis, pain, respiratory distress, or heart failure itself. Pathological tachyarrhythmias that should be differentiated include atrioventricular re‐entrant tachycardia, atrioventricular nodal re‐entrant tachycardia, ectopic atrial tachycardia, multifocal atrial tachycardia, junctional ectopic tachycardia, and AFL. When atrial activity is unclear on the surface ECG, diagnostic adenosine or ATP may be useful because transient atrioventricular block can unmask atrial activity and reveal flutter waves [11, 12]; however, clinicians should recognize that adenosine does not terminate AFL because the atrioventricular node is not part of the re‐entrant circuit.

When differentiating between ventricular dilatation and systolic dysfunction in such settings, the differential diagnosis includes myocarditis, primary dilated cardiomyopathy, metabolic or mitochondrial disorders, endocrine disorders, structural heart disease, and coronary artery abnormalities. In this case, the absence of fever or systemic illness, negative inflammatory and microbiological findings, the absence of structural heart disease on echocardiography, negative metabolic screening results, and the rapid recovery of left ventricular size and function following restoration of sinus rhythm strongly supported the diagnosis of TIC due to persistent AFL rather than primary cardiomyopathy or myocarditis.

While reports indicate that pediatric TIC generally improves within 2–6 months after arrhythmia control, it remains unclear how long tachyarrhythmia must persist before TIC develops [2]. Parental observations of intermittent irregular breathing suggest that AFL may have been present for several days. This difficulty in determining the onset of tachycardia is a common challenge highlighted in other pediatric TIC reports [4, 5, 10].

There is no established evidence‐based recommendation for routine anticoagulation after cardioversion of isolated infantile AFL in patients without structural heart disease or intracardiac thrombus [13]. In our institution, antithrombotic therapy for infantile AFL is individualized according to thrombotic risk factors such as intracardiac thrombus, structural or postoperative heart disease, severe ventricular dysfunction, marked chamber dilatation, central venous catheterization, or coagulation abnormalities. We used aspirin temporarily because severe but reversible LV dysfunction created a transient thrombotic‐risk state.

**FIGURE 3:**
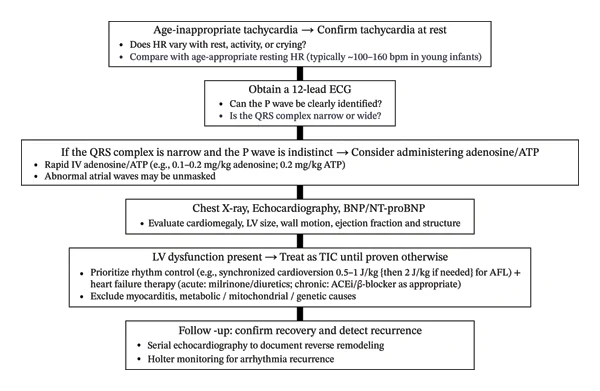
Practical algorithm for persistent age‐inappropriate tachycardia detected during routine infant health supervision. Confirm tachycardia at rest and obtain a 12‐lead ECG promptly; in narrow QRS tachycardia with unclear P waves, diagnostic adenosine/adenosine triphosphate (ATP) may unmask atrial activity and clarify the mechanism. If tachyarrhythmia persists or cardiomegaly is suspected, perform echocardiography and measure BNP/NT‐proBNP, and when left ventricular dysfunction is present, treat as tachycardia‐induced cardiomyopathy (TIC) until proven otherwise by prioritizing early rhythm control and heart failure therapy. ATP, adenosine triphosphate; BNP, B‐type natriuretic peptide; NT‐proBNP, N‐terminal pro‐B‐type natriuretic peptide.

From a practical standpoint, the most actionable message of this case is that persistent age‐inappropriate tachycardia at rest warrants a structured evaluation even when an infant appears clinically well. In routine health checkup, tachycardia may be attributed to crying or situational stress, potentially delaying recognition. We therefore provide a simple stepwise algorithm (Figure 3) that emphasizes 12‐lead ECG acquisition, diagnostic adenosine/ATP to unmask atrial activity in narrow QRS tachycardia with unclear P waves, and echocardiography with natriuretic peptide testing when tachyarrhythmia persists or cardiomegaly is suspected. When left ventricular dysfunction is present, the algorithm highlights the importance of considering TIC until proven otherwise and prioritizing early rhythm control alongside standard heart failure therapy.

## 4. Conclusions

TIC should be considered in infants with unexplained ventricular dysfunction when a tachyarrhythmia is present, because ventricular function can normalize after rhythm control. Even in apparently healthy infants, careful auscultation and heart rate measurement are essential components of health supervision. Persistent tachycardia at rest that is inappropriate for age should prompt ECG evaluation rather than being attributed to crying or anxiety. Early detection of tachyarrhythmias such as AFL may prevent progression to overt heart failure and improve outcomes.

## Author Contributions

Toshinobu Ifuku conceptualized and designed the study and drafted the initial manuscript. Fumito Wakamatsu and Keigo Nakatani reviewed the manuscript and provided conceptual advice. Toshinobu Ifuku and Fumito Wakamatsu treated patients and critically reviewed the manuscript.

## Funding

No funding was received for this manuscript.

## Disclosure

This manuscript has been prepared with attention to CARE case reporting guidelines. All authors read and approved the final manuscript as submitted and agreed to be accountable for all aspects of this work.

## Consent

Informed consent was obtained from the patient and his parents for the publication of this case report and accompanying images.

## Conflicts of Interest

The authors declare no conflicts of interest.

## Supporting Information

Additional supporting information can be found online in the Supporting Information section.

## Supporting information


**Supporting Information** 2026.4.19 CARE checklist.

## Data Availability

The data supporting the current study are available from the corresponding author upon request.
